# Erythrogram Patterns in Dogs with Chronic Kidney Disease

**DOI:** 10.3390/vetsci8070123

**Published:** 2021-06-30

**Authors:** Ilaria Lippi, Francesca Perondi, George Lubas, Eleonora Gori, Alessio Pierini, Alessandra D’Addetta, Veronica Marchetti

**Affiliations:** Department of Veterinary Science, University of Pisa, 56122 San Piero a Grado (Pisa), Italy; ilaria.lippi@unipi.it (I.L.); f.perondi87@gmail.com (F.P.); george.lubas@unipi.it (G.L.); pierini.alessio2004@gmail.com (A.P.); ale.daddetta@gmail.com (A.D.); veronica.marchetti@unipi.it (V.M.)

**Keywords:** dog, CKD, anemia, erythrogram

## Abstract

Anemia is considered a common finding in dogs with chronic kidney disease (CKD), typically as normochromic, normocytic, and non-regenerative. Although anemia can occur at any CKD IRIS (International Renal Interest Society) stage, its severity is related with the loss of kidney function. The aim of the present study was to retrospectively evaluate quantitative and morphological abnormalities of the erythrogram in dogs at different CKD IRIS stages. A total of 482 CBCs from 3648 initially screened were included in the study. Anemia was present in 302/482 (63%) dogs, in the majority of which it was normochromic, normocytic, and non-regenerative (295/302; 98%). The number of reticulocytes was <60,000/μL in the majority of dogs (248/295; 84%), with a correlation between poor regeneration rate and progression of CKD (*p* = 0.0001). The frequency of anemia significantly differed (*p* = 0.0001) among the IRIS stages: 108/231 (47%) in IRIS 2, 77/109 (71%) in IRIS 3, and 117/142 (82%) in IRIS 4. Dogs at IRIS stages 3 and 4 were more likely to have moderate to severe anemia, compared to dogs at IRIS stage 2 (*p* = 0.0001). Anisocytosis was the most frequent morphological abnormality (291/482; 60%), whereas the presence of poikilocytosis showed an association with progression of IRIS stages (*p* = 0.009). Among different morphological abnormalities, the frequency of fragmented red blood cells and Howell–Jolly bodies showed a significant association with the progression of CKD. Anemia was a frequent finding in CKD dogs, mostly associated with none to poor regeneration rate. Similar to human medicine, advanced CKD stages are more frequently characterized by morphological alterations, such as fragmented red blood cells and Howell–Jolly bodies, which may suggest a more severe condition of reduced bone marrow activity and microangiopathy.

## 1. Introduction

Anemia is considered a common finding in dogs with chronic kidney disease (CKD) [1], which typically occurs as normochromic, normocytic, and non-regenerative [2]. Although no published data are available regarding anemia in CKD dogs at different stages, clinical experiences seem to show an association between frequency of anemia and progression of CKD. Anemia in CKD dogs is mainly related to the decreased production of erythropoietin as well as other mechanisms, such as inflammation, iron deficiency or inappropriate utilization, decreased red blood cell (RBC) survival due to metabolic or mechanical injuries, and the anti-proliferative effects of uremic toxins [3]. The severity of anemia has been reported to correlate positively with serum creatinine concentrations [1] and to become clinically relevant in patients with CKD International Renal Interest Society (IRIS) stages 3 and 4 [4]. Morphological abnormalities of RBCs, such as spiculated (echinocyte) and deformed RBCs (acanthocytes), have been occasionally reported in the blood smear evaluation of CKD dogs, although their association with the severity of CKD has not been investigated [2]. 

The aim of the present study was to retrospectively evaluate quantitative and morphological abnormalities of erythrogram in dogs at different CKD IRIS stages.

## 2. Materials and Methods

### 2.1. Case Selection

Medical records of CKD dogs, presented to the Veterinary Teaching Hospital “Mario Modenato” of Pisa University, between January 2014 and December 2020, were retrospectively evaluated. Inclusion criteria for CKD included complete case-log with history, and laboratory and ultrasonographic findings consistent with CKD. Specifically, diagnosis of CKD was based on chronic history of azotemia (more than 3–4 months), progressive weight loss, poor appetite, polyuria–polydipsia (PU/PD), and renal ultrasound findings of irregular shape, reduced cortico-medullary distinction, and hyperechoic cortices [5]. 

Dogs were excluded if (1) historical, laboratory, and/or ultrasonographic findings were consistent with acute kidney injury (AKI); (2) there was a unavailable report of the blood smear evaluation; (3) use of alpha-darbepoetin or red blood cell transfusion prior to the presentation; (4) severe comorbidities, which might affect erythrogram, such as hyperadrenocorticism, hypothyroidism, and neoplasia; and (5) finally, since the same dog was included in the case selection only once, rechecks of the same patients were excluded. 

Included dogs were classified according to the IRIS guidelines for CKD, based on serum creatinine concentration as follows: stage 2 (serum creatinine 1.4 to 2.8 mg/dL); stage 3 (serum creatinine 2.9 to 5.0 mg/dL); and stage 4 (serum creatinine > 5.0 mg/dL) [6].

### 2.2. Hematological Parameters 

Complete blood counts (CBCs) were performed using a laser cell counter (Procyte DX, IDEXX Laboratories, Westbrook, ME, USA), and a blood smear stained with May–Grünwald Giemsa (Aerospray Wescor, Delcon, Milan, Italy) was microscopically examined by experienced and trained clinical pathologists. For each dog included in the study, the following parameters were evaluated: red blood cell count (RBC), hematocrit (HCT) and hemoglobin (HGB) values, mean corpuscular volume (MCV), mean corpuscular hemoglobin (MCH), mean corpuscular hemoglobin concentration (MCHC), red blood cell distribution width (RDW), and absolute reticulocytes count (RET). Anemia was considered mild if the HCT range was 37–30%, moderate if the HCT range was 29–20%, and severe if the HCT was below 19% [7,8]. Anemia was considered microcytic for MCV < 61 fL, normocytic for MCV between 61 and 73 fL, and macrocytic for MCV > 73 fL. Anemia was considered hypochromic for MCHC < 32 g/dL, normochromic for MCHC between 32 and 38 g/dL, and hyperchromic for MCHC > 38 g/dL. Regeneration rate was considered absent for RET < 60,000/μL, mild for RET ranging between 61,000 and 150,000/μL, and moderate for RET > 150,000/μL [7]. The degree of the different morphological abnormalities found on RBCs (poikilocytosis) at the microscopic evaluation was assessed according to Weiss (1984) as follows: weak ranging from −/+ to 1+, moderate for 2+, and marked ranging from 3+ to 4+ [9].

Serum creatinine and urea were assessed in liquid chemistry (instrument SAT450 or Liasys, with dedicated reagent kits, Analyzer Medical System—AMS, Rome, Italy).

### 2.3. Statistical Analysis

Continuous variables were tested for normality through the Kolmogorov–Smirnov test. 

Kruskal–Wallis test and Dunn’s multiple comparisons test were used to compare CBC parameters among different CKD groups (IRIS 2, IRIS 3, and IRIS 4). Correlation analysis between RBC, HCT, and HGB values, and serum creatinine and urea was performed using Spearman’s correlation test. Chi squared test of independence was used to compare the frequency and the degree of anemia, the frequency of different types of anemia according to MCV and MCHC, and the frequency and the degree of different morphological abnormalities of erythrograms among dogs at different stages of CKD. Statistical analysis was performed using commercial statistical software (IBM, SPSS v.25, IBM Corp., New York, NY, USA), and all data were considered statistically significant for *p* value < 0.05.

## 3. Results

The retrospective medical record evaluation of electronic clinical database of the University of Pisa Veterinary Teaching Hospital gave initially a total of 3648 azotemic dogs within the selected time interval. Afterwards, among the initial case-load, 3166 dogs were excluded due to the following reasons: 441 dogs had AKI, 2511 were rechecks of the same dog, in 127 dogs the blood smear evaluation was unavailable, and 87 dogs were previously treated with alpha-darbepoetin or blood transfusion. Finally, a total of 482 dogs with CKD were included in the study. 

Of the 482 enrolled dogs, 231 dogs (47.9%) were in CKD IRIS stage 2, 109 dogs (22.6%) were in IRIS stage 3, and 142 dogs (29.5%) were in IRIS stage 4. According to sex, 214 dogs (44.4%) were intact males, 20 dogs (4.1%) were neutered males, 159 dogs (32.9%) were intact females, and 89 dogs (18.6%) were spayed females. Median age was 9.6 years (range 5.9–12.4 years), and the most represented breeds were mix-breed (*n* = 145; 30%), Boxers (*n* = 31; 6.4%), Labrador retrievers (*n* = 30; 6.2%), German shepherds (*n* = 20; 4.1%), and Golden Retrievers (*n* = 17; 3.5%).

Anemia was present in 302/482 dogs (63%). The frequency of anemia was of 47% (108/231) in IRIS 2, 71% (77/109) in IRIS 3, and 82% (117/142) in IRIS 4. A Chi squared test showed a statistical association between frequency of anemia and progression of the IRIS stage (*p* = 0.0001). Characterization of anemia according to MCV and MCHC in dogs at different IRIS stages is reported in Table 1. Normochromic and normocytic anemia was the most frequent type of anemia, which was found in 209/302 (69%) dogs. According to the regeneration rate, the majority of dogs (239/302; 79%) showed non-regenerative anemia (RET < 60,000/μL) (Table 2).

Median values of erythrogram parameters of dogs at different CKD IRIS stages are reported in Table 3.

**Figure 1:**
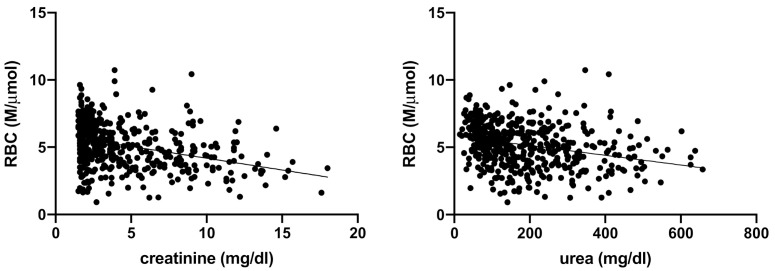
Spearman’s correlation analysis between RBC and serum creatinine and urea. Statistically significant, negative linear correlation (*p* < 0.0001; r −0.3470) between RBC (M/μmol) and serum creatinine (mg/dL), and statistically significant negative linear correlation (*p* < 0.0001; r −0.2840) between RBC (M/μmol) and serum urea (mg/dL) in the totality of anemic CKD dogs (*n* = 302).

**Figure 2:**
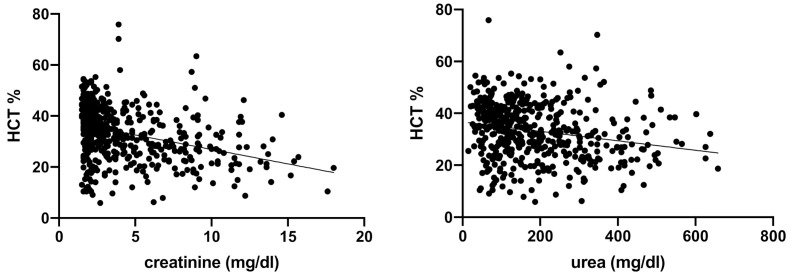
Spearman’s correlation analysis between HCT and serum creatinine and urea. Statistically significant, negative linear correlation (*p* < 0.0001; r −0.3439) between HCT (%) and serum creatinine (mg/dL), and statistically significant negative linear correlation (*p* < 0.0001; r −0.2194) between HCT (%) and serum urea (mg/dL) in the totality of anemic CKD dogs (*n* = 302).

**Figure 3:**
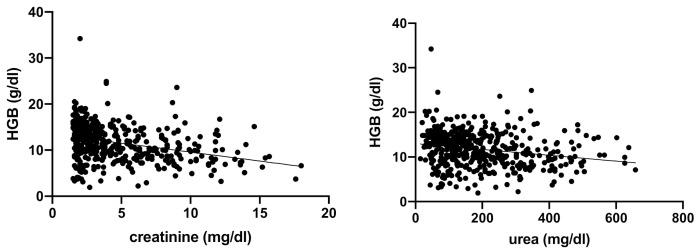
Spearman’s correlation analysis between HGB and serum creatinine and urea. Statistically significant, negative linear correlation (*p* < 0.0001; r −0.3286) between HGN (g/dL) and serum creatinine (mg/dL), and statistically significant negative linear correlation (*p* < 0.0001; r −0.2194) between HGB (g/dL) and serum urea (mg/dL) in the totality of anemic CKD dogs (*n* = 302).

Spearman’s correlation analysis showed a negative, linear correlation between serum urea and creatinine and the following erythrogram parameters: RBC, HCT, and HGB (respectively, Figure 1, Figure 2 and Figure 3).

Morphological abnormalities of the erythrogram are reported in Table 4. 

## 4. Discussion

In our population of CKD dogs, anemia was a very common finding, which was highlighted in approximatively 60% of dogs, especially in more advanced IRIS stages. The frequency of anemia increased particularly in dogs at IRIS stages 3 and 4, in which the frequency exceeded 70%. This finding is in agreement with previous reports in human medicine, where the frequency of anemia increases with the progression of CKD stage, reaching values of 70–90% in end stage renal disease [10,11]. The main mechanism of anemia in CKD has been historically recognized in erythropoietin (EPO) deficiency, which is responsible for poor maturation and differentiation of RBC precursors. Beside EPO deficiency, other causes of anemia have been progressively identified in CKD patients. Impaired response of the bone marrow to EPO may occur as a consequence of the action of circulating uremic toxins, inflammation, vitamin B deficiency, and/or decreased availability of iron for erythropoiesis [11]. The lifespan of RBCs in uremic patients is usually shorter than normal, due to the effects of the metabolic and/or vascular environment on RBC survival [3,12].

Although anemia seems to be more frequent in dogs with advanced stages of CKD, dogs at IRIS stage 2 show a frequency close to 47%. Therefore, it is plausible that anemia develops at early stages of CKD, similarly to humans [10]. People with CKD tend to develop anemia at early stages of CKD, although the severity of anemia and need for treatment increase with the progression of the disease [10]. In our study, IRIS stage 3 and 4 are characterized by a higher number of dogs with moderate to severe degrees of anemia compared to IRIS stage 2. Similar to human medicine, dogs at advanced IRIS stages are more likely to show moderate to severe anemia. 

Evidence of the association between anemia and progression of renal disease has been previously reported in dogs [1,2,4]; however, no data concerning the frequency of anemia in dogs at different stages of CKD are currently available. One prospective study including 17 dogs with CKD reported a normocytic, normochromic anemia in 71% of dogs [1], while a report of juvenile nephropathy in 37 Boxer dogs found anemia in 44% of cases, but the anemia was not characterized [13,14]. 

In agreement with previous reports [2], the majority of dogs in our study showed no to mild regeneration. Anemia was non regenerative in 79% of dogs, and the frequency of low reticulocyte count (<60,000/μL) was strongly associated with the progression of the IRIS stage. Dogs at IRIS stages 3 and 4 were more likely to show no to poor regeneration, compared to IRIS stage 2. 

The primary mechanism related to anemia in CKD dogs is decreased renal production of EPO [2]. EPO is produced in renal peritubular fibroblast-like type-1 interstitial cells in response to cellular hypoxia [1,2]. EPO stimulates red blood cell production in the bone marrow, by enhancing survival of certain erythrocyte progenitor cells [2]. Non-regenerative anemia has been often reported in dogs with CKD [2], and in these patients, bone marrow aspirates usually revealed hypoplasia of the erythroid precursors, with little or no interference with normal leukopoiesis and megakaryocytopoiesis [2]. Therefore, it is plausible that EPO deficiency may be a leading cause of poor regeneration in our cohort of dogs. We unfortunately did not evaluate EPO values in any of these dogs, as the technique to assess this stimulating factor for erythroid pool is not currently validated, and the EPO values are variable in anemic and polycythemic patients [15]. During a regenerative response, increased RDW values and reticulocytosis are generally observed. Healthy and adult dogs normally release aggregate reticulocytes from the bone marrow (usually ≤ 1.0%), which then complete maturation in roughly 24 h [16]. In our study, both median RDW and reticulocytes significantly decreased with the progression of the IRIS stage, confirming the poor regeneration of the disease. This finding is also reinforced by the lack of increase of MCV, in response to the worsening of anemia. In physiological conditions, MCV tends to increase in the presence of regeneration, indicating the release into the circulation of immature reticulocytes and/or erythrocytes larger than mature ones. In our cohort, no significant increase in the median MCV was noticed in IRIS stage 3 and 4, despite the worsening of anemia. 

Another confirmation of the reduced regeneration process is the lower frequency of polychromasia with the progression of CKD. The finding of a higher degree of polychromasia in IRIS stage 2 compared to stages 3 and 4 may reflect more intense activity of the bone marrow in the earlier stages. Polychromasia is commonly associated with a premature release of immature forms of RBCs. It is plausible that the progression of CKD depresses the activity of the bone marrow, reducing the release of immature RBCs.

Typically, the severity of anemia is roughly proportional to the loss of kidney function [2,17], as reported by the study of King et al. (1992), where the hematocrit correlated negatively with serum creatinine concentrations [1]. Our findings seemed to confirm human data, as RBC, HCT, and HGB showed a negative linear correlation with serum creatinine and urea. The worsening of anemia with the progression of the IRIS stage may be related to several mechanisms beside EPO deficiency. Serum of uremic patients has been associated with inhibition of hematopoietic progenitor growth. Among these factors, the uremic toxin indoxyl sulphate may play a significant role, interfering with erythropoiesis and limiting EPO gene transcription during hypoxia. On the other hand, indoxyl sulphate may also shorten RBC survival by triggering suicidal erythrocyte death [10]. 

According to morphological abnormalities, anisocytosis was the most frequent finding found. Although anisocytosis was found in the 60% of the dogs of the present study, its frequency and degree were not associated with the progression of the disease. Moreover, the median RDW of our dogs was within the reference range and did not change significantly among the IRIS stages. Anisocytosis is a parameter commonly associated with a high variability of the sizes of RBCs, which has been historically associated with renal failure [18,19]. In the course of anemia, the finding of anisocytosis, as well as elevated RDW, may reflect different pathological conditions, such as deficiency of iron, folic acid, and vitamin B12, or inflammation, as well as hemolytic uremic syndrome [20]. Although no information on the iron panel was available in our study, a potential role of iron metabolism impairment on the frequency of anisocytosis cannot be rule-out. As is known, systemic iron balance is maintained by regulating dietary iron absorption and iron release from storage sites. In CKD, iron metabolism may be significantly affected, on one hand, by ongoing iron losses due to chronic bleeding, frequent blood samples, and losses during dialysis sessions; and on the other hand, by a reticuloendothelial cell iron blockade. In particular, CKD patients may present a significantly elevated serum concentration of hepcidin, which is responsible for impaired intestinal absorption of iron, and reticuloendothelial cell iron blockade [21]. 

In our cohort of dogs, poikilocytosis was found in 28% of the CKD population, and its frequency increased significantly in dogs at IRIS stages 3 and 4. Echinocytes were the most frequent form of poikilocytosis in our population (53%), followed by fragmented red cells (39%) and acanthocytosis (34%). Interestingly, acanthocytosis has been previously reported as one of the most frequent abnormalities in rabbits with renal failure [22]. Although the finding of echinocytes has also been associated with non-pathological conditions (such as prolonged blood storage, artifacts due to excessive EDTA, or slow drying of the smear), its association with acanthocytosis may be related to cholesterol enrichment of the RBC membrane. Elevated serum cholesterol concentrations would be responsible for formation of RBC membrane projections and increased membrane rigidity. As dyslipidemia and elevated serum cholesterol are very common findings in human CKD, it is possible that they may a play a key role also in CKD dogs [22,23]. The authors’ unpublished clinical experience reported a frequency of hypercholesterolemia and hypertriglyceridemia of approximately 70–80% of CKD dogs. Interestingly, in our cohort of dogs, the frequency of fragmented red cells increased significantly with the progression of the IRIS stage. The finding of fragmented red cells together with acanthocytes is not surprising, as fragmented RBC may arise from the fragmentation process of acanthocytes. Physical damage of RBC and fragmentation have been documented in dogs with glomerulonephritis, due to microangiopathy. Turbulent blood flow, related to endothelial fibrin deposition and microthrombi, may promote microangiopathic damage [24]. Uremic toxins may also play a significant role in promoting RBC fragmentation by increasing the inlet of ionized calcium and the outlet of phosphatidylserine in the lipid bilayer, and by increasing the cellular rigidity [22]. 

Howell–Jolly bodies (HJBs) were another morphological abnormality found in our cohort, which frequency increased with the progression of CKD stage. On the other hand, polychromasia was significantly reduced from stage 2 to stage 4. The presence of HJBs during renal failure may be caused by a possible alteration of the blood–bone–marrow barrier, due to the presence of uremic toxins. The increased number of HJBs in advanced stages of CKD may also be related to a condition of systemic inflammation and elevated oxidative damage, in which the removal of HJBs from the bloodstream is reduced due to the lower phagocytosis activity of macrophages [18,25].

Given the association between progression of CKD and severity of anemia, a prospective study evaluating the role of uremic toxins (such as indoxyl sulphate) in anemic CKD dogs would be advisable. In particular, the serum levels of uremic toxins may be compared to serum markers of systemic inflammation, as well as oxidative damage. Prospective studies should also investigate factors that may affect RBC membrane integrity, such as the lipid profile. 

The present study has several limitations. First of all, the retrospective nature of the study over a wide time interval did not allow us to include IRIS stage 1 dogs. Serum SDMA has been only recently introduced in the routine blood work screening of CKD. Therefore, it is possible that dogs with IRIS stage 1 were undiagnosed prior to SDMA evaluation. As the number of dogs in IRIS stage 1 was significantly lower that the number of dogs in the other stages, we opted not to include them in the statistical analysis. Unfortunately, iron profiles were available only in a limited number of dogs, which did not allow us to include iron profiles in the statistics. Finally, although the study population was selected base on the presence of uremia due to CKD, it was not possible to recognize the presence of concomitant comorbidities as they can also affect the erythrogram profile.

## 5. Conclusions

In conclusion, anemia seems a very frequently found disorder in CKD dogs, mostly associated with poor regeneration rate. Although anemia may be present at any CKD IRIS stage, the frequency of this hematological disorder and its degree of severity seem to be proportional to the loss of kidney function, with low reticulocyte count (<60,000/μL) associated with the progression of the IRIS stage. Similar to human medicine, advanced CKD IRIS stages are more frequently characterized by RBC morphological alterations, such as fragmented red blood cells and Howell–Jolly bodies, which may suggest a more severe condition of reduced bone marrow activity and microangiopathy.

## Figures and Tables

**Table 1:** Chi squared comparison of different types of anemia among dogs at different CKD IRIS stages.

	IRIS 2 (*n* = 108)	IRIS 3 (*n* = 77)	IRIS 4 (*n* = 117)	*p*-Value
**Frequency of different types of anemia**				0.203
Microcytic–Hypochromic	1/108 (1%)	2/77 (3%)	0/117 (0%)	
Microcytic–Normochromic	26/108 (24%)	11/77 (14%)	22/117 (19%)	
Microcytic–Hyperchromic	1/108 (1%)	3/77 (4%)	6/117 (5%)	
Normocytic–Normochromic	74/108 (69%)	57/77 (74%)	78/117 (67%)	
Normocytic–Hyperchromic	2/108 (2%)	0/77 (0%)	2/117 (2%)	
Macrocytic–Hypochromic	3/108 (3%)	0/77 (0%)	3/117 (3%)	
Macrocytic–Normochromic	1/108 (1%)	4/77 (5%)	6/117 (5%)	

**Table 2:** Chi squared comparison of the frequency and degree of anemia and regeneration rate among dogs at different CKD IRIS stages.

	IRIS 2	IRIS 3	IRIS 4	*p-*Value
**Frequency of anemia**				*0.0001*
Anemia	(*n* = 108) 108/231 (47%)	(*n* = 77) 77/109 (71%)	(*n* = 117) 117/142 (82%)	
**Degree of anemia**				*0.0001*
Mild (HCT 37–30%)	(*n* = 104) 49/104 (47%)	(*n* = 76) 34/76 (45%)	(*n* = 115) 27/115 (23%)	
Moderate (HCT 29–20%)	34/104 (33%)	34/76 (45%)	62/115 (54%)	
Severe (HCT < 19%)	21/104 (20%)	8/76 (10%)	26/115 (23%)	
**Degree of regeneration**				*0.0001*
None (RET < 60,000/μL)	(*n* = 104) 78/104 (75%)	(*n* = 76) 61/76 (80%)	(*n* = 115) 109/115 (95%)	
Mild (RET 60,000–150,000/μL)	20/104 (19%)	12/76 (16%)	5/115 (4%)	
Moderate (RET > 150,000/μL)	6/104 (6%)	3/7 (4%)	1/115 (0.8%)	

Degree of anemia was defined as mild (HCT 37–30%), moderate (HCT 29–20%), and severe (HCT < 19%); degree of regeneration was defined as none (RET < 60,000/μL), mild (RET 60,000–150,000/μL), and moderate (RET > 150,000/μL).

**Table 3:** Kruskal-Wallis comparison of erythrogram parameters of dogs at different IRIS stages.

Parameter	Reference Range	CKD (*n* = 482)	IRIS 2 (*n* = 231)	IRIS 3 (*n* = 109)	IRIS 4 (*n* = 142)	*p*-Value
RBC (M/µL)	5.6–8.8	5.17 ± 1.64	5.86 (4.76–6.7)	5.0 (4.0–6.0)	4.2 (3.3–5.2)	*<0.0001*
HCT (%)	37.3–61.7	33.2 ± 10.7	37.6 (30.4–43.9)	32.1 (26.0–39.2)	27.3 (21.2–335.0)	*<0.0001*
HGB (g/dL)	13.1–20.5	11.9 (9.2–14.4)	13.3 (10.8–15.2)	11.2 (13.8–9.5)	9.8 (12.1–7.7)	*<0.0001*
MCV (fL)	61.6–73.5	64.6 (61.5–67.5)	64.8 (61.6–67.2)	64.8 (61.8–68.0)	64 (60.8–67.7)	0.53
MCH (pg)	21.2–25.9	22.8 (21.8–23.7)	22.6 (21.6–23.6)	23 (22.0–23.7)	23 (21.9–24)	0.11
MCHC (g/dL)	32.0–37.9	35.3 (34.1–36.3)	35.1 (34.0–35.9)	35.3 (33.9–36.4)	35.7 (34.5–36.7)	*0.0002*
RDW (%)	13.6–21.7	16.2 (14.9–18.2)	16.5 (15.0–18.8)	16 (14.7–18.3)	15.8 (14.7–17.7)	*0.024*
RET (K/µL)	10–110	24 (1–166)	34 (3–166)	23 (12–56)	16 (1–78)	*0.003*

RBC, red blood cells; HCT, hematocrit; HGB, hemoglobin; MCV, mean corpuscular volume; MCH, mean cell hemoglobin; MCHC, mean corpuscular hemoglobin concentration; RDW, red blood cell distribution width; RET, reticulocyte number. CKD anemic dogs (*n* = 482) were distributed as IRIS 2 (*n* = 231), IRIS 3 (*n* = 109), and IRIS 4 (*n* = 142). Normally distributed data are represented as mean ± standard deviation, and non-normally distributed data are presented as median and range. The majority of dogs at IRIS stage 2, 3, and 4 showed normocytic–normochromic anemia. No statistically significant difference in the frequency of different types of anemia was found at different IRIS stages.

**Table 4:** Chi squared comparison of morphological abnormalities of the erythrogram in dogs at different CKD IRIS stages.

	Degree	IRIS 2	IRIS 3	IRIS 4	*p-*Value
Anisocytosis (291/482; 60%)	weak	90	45	56	0.77
	moderate	40	15	25	
	marked	7	5	8	
Polychromasia (113/482; 23%)	weak	59	1	18	*0.001*
	moderate	9	1	2	
	marked	1	1	1	
Howell–Jolly bodies (87/482; 18%)	weak	30	11	31	0.186
	moderate	7	0	5	
	marked	3	0	0	
Poikilocytosis (135/482; 28%)	weak	33	23	29	0.315
	moderate	15	14	7	
	marked	3	4	7	
Echinocyte (73/135; 54%)	weak	16	11	17	0.464
	moderate	7	9	12	
	marked	0	1	0	
Fragmented cells (54/135; 40%)	weak	21	7	20	*0.008*
	moderate	2	4	0	
	marked	0	0	0	
Acantocytes (45/135; 33%)	weak	15	5	15	0.05
	moderate	3	5	2	
	marked	0	0	0	
Dacriocytes (13/135, 10%)	weak	4	8	0	0.488
	moderate	0	1	0	
	marked	0	0	0	
Eccentrocytes (5/135; 4%)	weak	1	1	0	0.441
	moderate	0	0	1	
	marked	1	0	1	

## Data Availability

Data supporting reported results can be found on the electronic database (OCIROE) of the Vet-erinary Teaching Hospital of the University of Pisa.
